# Synthesis and modification of uniform PEG-neridronate-modified magnetic nanoparticles determines prolonged blood circulation and biodistribution in a mouse preclinical model

**DOI:** 10.1038/s41598-019-47262-w

**Published:** 2019-07-24

**Authors:** Vitalii Patsula, Daniel Horák, Jan Kučka, Hana Macková, Volodymyr Lobaz, Pavla Francová, Vít Herynek, Tomáš Heizer, Petr Páral, Luděk Šefc

**Affiliations:** 1 Institute of Macromolecular Chemistry, Czech Academy of Sciences, Heyrovského nám. 2, 162 06 Prague 6, Czech Republic; 2 Center of Advanced Preclinical Imaging, First Faculty of Medicine, Charles University, Salmovská 3, 120 00 Prague 2, Czech Republic

**Keywords:** Materials science, Nanoscience and technology

## Abstract

Magnetite (Fe_3_O_4_) nanoparticles with uniform sizes of 10, 20, and 31 nm were prepared by thermal decomposition of Fe(III) oleate or mandelate in a high-boiling point solvent (>320 °C). To render the particles with hydrophilic and antifouling properties, their surface was coated with a PEG-containing bisphosphonate anchoring group. The PEGylated particles were characterized by a range of physicochemical methods, including dynamic light scattering, transmission electron microscopy, thermogravimetric analysis, Fourier transform infrared spectroscopy, and magnetization measurements. As the particle size increased from 10 to 31 nm, the amount of PEG coating decreased from 28.5 to 9 wt.%. The PEG formed a dense brush-like shell on the particle surface, which prevented particles from aggregating in water and PBS (pH 7.4) and maximized the circulation time *in vivo*. Magnetic resonance relaxometry confirmed that the PEG-modified Fe_3_O_4_ nanoparticles had high relaxivity, which increased with increasing particle size. In the *in vivo* experiments in a mouse model, the particles provided visible contrast enhancement in the magnetic resonance images. Almost 70% of administrated 20-nm magnetic nanoparticles still circulated in the blood stream after four hours; however, their retention in the tumor was rather low, which was likely due to the antifouling properties of PEG.

## Introduction

The previous several decades of continuous research in the field of nanotechnology has resulted in the development of a variety of nanomaterials with specially tailored properties, allowing their application in biomedicine^1,2^. Among them, magnetic nanoparticles are distinct due to their unique magnetic properties, large surface-to-volume ratio, small size, and ability to function at the cellular level. These characteristics make them appropriate candidates for medical applications, such as contrast agents for magnetic resonance imaging (MRI), magnetically guided carriers for drug delivery, or heat mediators for hyperthermia^3–5^. Magnetic nanomaterials, which incorporate metallic, bimetallic, and metal oxide nanoparticles^6^, in particular, maghemite (γ-Fe_2_O_3_) and magnetite (Fe_3_O_4_), are advantageous due to their low toxicity^7^, biocompatibility and the possibility of easily modifying the surface with polymer shells, drugs and/or targeting and imaging ligands^8,9^. In organism, intravenously administrated iron oxide nanoparticles are recognized by mononuclear phagocytic system depending on the size and transported to organs, predominantly liver and spleen (d > 8 nm), or filtered via kidneys (d < 8 nm)^10^. Borderline in size excluded by kidneys is not sharp; even 10 or 14 nm particles were found in urine^11,12^. At the cellular level, mainly liver sinusoidal endothelial cells, Kupffer cells, and splenic red pulp macrophages gradually degrade iron oxides in the lysosomes^13^, subsequently transferring Fe to endogenous iron storing proteins^14^. After intravascular administration, particles should be colloidally stable at a high ionic strength, which can induce particle agglomeration and trigger recognition and clearance by the reticuloendothelial system. Consequently, physicochemical properties, i.e., size, polydispersity, geometry, charge, and surface coating are critical parameters strongly determining ability of the particles to circulate in the blood stream. These characteristics can be controlled by the selection of a proper method for nanoparticle preparation. Currently, many approaches for iron oxide synthesis have been developed to improve the control of particle morphology and surface properties. These methods include coprecipitation, microemulsion, sol-gel, flame spray pyrolysis, thermal decomposition, etc.^15–17^. The latter technique has a significant advantage because it allows the preparation of uniform particles with excellent control over their size and magnetism. A defined particle size, together with uniformity, can provide more precise and reproducible *in vivo* results than particles with a broad size distribution^12^. However, the significant disadvantage of this method is the hydrophobic nature of the particle coating, which requires additional postsynthetic modifications, e.g., ligand exchange to make the particles water-dispersible and applicable in biomedicine.

Earlier publications described superior results obtained with poly(ethylene glycol) (PEG)-based coating of iron oxides attributed to its hydrophilicity, biocompatibility, non-antigenicity, and antifouling properties, which remarkably extend circulation of nanoparticles in blood^12^. However, the behavior of PEGylated particles strongly depends on the molecular weight (*M*_w_) of PEG and its architecture and surface density that affect the chain conformation at the surface^18,19^. Generally, stealth properties of nanoparticles in the blood stream are achieved by highly dense PEG shells with *M*_w_ = 2–10 kg/mol^19^. The conjugation of macromolecules to particle surfaces can be achieved by anchoring groups capable of strong interactions with iron, e.g., aromatic vicinal diols, carboxyls, bisphosphonates, or hydroxamates^20–23^; the strongest bonding is achieved with bisphosphonate groups^24,25^. In particular, polymers containing hydroxybisphosphonate anchoring groups provide magnetite or lanthanide nanoparticle dispersions that are colloidally stable under physiological conditions^26–28^. Such polymers are exemplified by PEG with hydroxybisphosphonate terminal moieties prepared via EDC/NHS chemistry or chloranhydride^29^. PEGylated iron oxide particles also increase the blood circulation time. For example, poly(maleic anhydride-*alt*-1-octadecene)-PEG-coated iron oxide provided blood half-life 105 min in mice^30^, PEGylated-arginin-coated iron oxide had at least 2 h circulation time^31^, and also (3-aminopropyl)triethoxysilane-PEG-functionalized or dopamine-PEG-coated magnetite escaped from the uptake by reticular endothelial system^32,33^.

The aim of this work was to prolong the circulation of iron oxide nanoparticles in the blood stream by optimizing their physiochemical parameters, compare them with literature^27,30,34^ and utilize the particles for passive tumor targeting via the enhanced permeability and retention (EPR) effect in a mouse model. We assume that this behavior will be beneficial especially for drug delivery systems, where drug can be released from the particles by changing pH. Differently sized magnetic nanoparticles were synthetized by a thermal decomposition method. To achieve superior colloidal stability of the particles in aqueous media, their surface was modified with PEG terminated by bisphosphonate anchoring groups (neridronate). The nanoparticles were characterized by various physicochemical methods to analyze the size, relaxivity, magnetization, and PEG surface density, and blood circulation time was determined in a mouse model. The particle accumulation in the tumor via EPR effect was also investigated.

## Experimental

### Materials

Octadec-1-ene (OD, 99%), icosane (IS, 99%), and FeCl_3_·6H_2_O (98%) were purchased from Sigma-Aldrich (St. Louis, MO, USA). Oleic acid (OA; 95%), hexane (99%), dichloromethane (99.9%), petroleum ether (b.p. 40–60 °C) ethanol (99%), NaOH (98.6%), HCl (35%), and salts (Na_2_HPO_4_·12∙H_2_O, KH_2_PO_4_, and NaCl) used for the preparation of 0.1 M phosphate buffer saline (PBS) were obtained from Lach-Ner (Neratovice, Czech Republic). α-Methoxy poly(ethylene glycol) succinimidyl ester (PEG-NHS; *M*_w_ = 2000 g/mol) was purchased from Rapp Polymere (Tuebingen, Germany). Cellulose dialysis membrane (14 kg/mol) was obtained from Spectrum Europe (Breda, Netherlands). Bovine serum albumin fraction V (BSA; *M*_w_ = 67 kg/mol) was purchased from Serva Electrophoresis (Heidelberg, Germany). Isoflurane was obtained from Baxter (San Juan, Puerto Rico). Fe(III) oleate and mandelate were prepared according to previous publication^23^. Additionally, 6-amino-1-hydroxy-1,1-hexanediyl)bis(phosphonic acid) monosodium salt (neridronate) was prepared according to a previously published report^26^. Ultrapure Q-water that was ultrafiltered using a Milli-Q Gradient A10 system (Millipore; Molsheim, France) was used in all experiments. All other reagent grade chemicals were purchased from Sigma-Aldrich and used as received.

### Synthesis of Fe_3_O_4_ nanoparticles

Monodisperse 10-nm Fe_3_O_4_ nanoparticles were synthesized by the thermal decomposition of Fe(III) oleate at 320 °C. Briefly, Fe(III) oleate (5.76 g) and OA (4.55 g) were dissolved in OD (40 ml) and the mixture was preheated at 125 °C for 60 min, and then heated at 320 °C for 30 min. The 20 and 31 nm Fe_3_O_4_ particles were obtained by the thermal decomposition of Fe(III) mandelate (3.26 g) in the presence of OA (4.4 g) in OD (40 ml) at 320 °C for 30 min or in IS (40 ml) at 343 °C for 60 min, respectively. The reaction was cooled to room temperature (RT), and the particles were precipitated by ethanol (100 ml) and magnetically separated. To remove the residual solvents and OA, the particles were washed with hot ethanol five times (~70 °C; 50 ml for each wash) and finally redispersed in toluene under Ar and stored.

### Synthesis of PEG-neridronate (PEG-Ner)

Neridronate (1.36 g) was dissolved in 0.5 M phosphate buffer (3 ml; pH 7.3) at 0 °C, the pH of the solution was maintained at 7.3 by adding 4 M aqueous NaOH, PEG-NHS (1 g) was added, and the reaction mixture was stirred at 0 °C for 6 h and then at RT for 16 h. After the reaction was completed, the pH was adjusted to 6 by adding 4 M HCl, PEG-Ner was extracted with CH_2_Cl_2_ (3 × 8 ml), the organic layers were united, and the solution was filtered through a 0.45 µm filter. CH_2_Cl_2_ was removed on a rotary evaporator at 30 °C, and the solid residue was dissolved in water (5 ml). To retrieve the phosphate groups on PEG-Ner, the aqueous solution was passed through an ion-exchange column packed with sulfonated polystyrene resin. Water was removed from the solution using a rotary evaporator at 60 °C (2.5 kPa) and PEG-Ner was finally vacuum-dried at 60 °C over phosphorus pentoxide.

### Synthesis of PEG-modified Fe_3_O_4_ nanoparticles (Fe_3_O_4_@PEG-Ner)

PEG-Ner (150 mg) was added to Fe_3_O_4_ nanoparticles (50 mg) in toluene/1,4-dioxane (3:2 v/v; 10 ml), the mixture was sonicated (UP200S Hielscher Ultrasound Technology; Teltow, Germany) for 5 min at 100 W, purged with Ar for 15 min, and stirred (900 rpm) at 70 °C for 48 h. The resulting PEG**-**modified Fe_3_O_4_ particles (Fe_3_O_4_@PEG-Ner) were precipitated three times with petroleum ether (80 ml for each precipitation), separated by a magnet, redispersed in water, and dialyzed against water for 48 h using a cellulose dialysis membrane (14 kg/mol). The final Fe_3_O_4_@PEG-Ner concentration reached ∼4.5 mg/ml.

### Nanoparticle characterization

Transmission electron microscopy (TEM) micrographs of the particles were obtained with a Tecnai G2 Spirit Twin 12 transmission electron microscope (TEM; FEI; Brno, Czech Republic). The number-average diameter (*D*_n_ = Σ N_i_*∙D*_i_/Σ N_i_), weight-average diameter (*D*_w_ = Σ N_i_*∙D*_i_^4^/Σ N_i_*∙D*_i_^3^) and dispersity (*Ð = D*_w_/*D*_n_) were determined by the measurement of at least 600 particles from TEM micrographs using Atlas software (Tescan Digital Microscopy Imaging; Brno, Czech Republic); N_i_ is the number and *D*_i_ is the diameter of i^th^ particle. The hydrodynamic diameter *D*_h_, polydispersity (*PI*) and electrophoretic mobility of the magnetic nanoparticles, which was converted to ζ-potential using the Smoluchowski equation, were obtained by dynamic light scattering (DLS) using a ZEN 3600 Zetasizer Nano instrument (Malvern Instruments; Malvern, UK) at RT. The *D*_h_ was calculated from the intensity-weighted distribution function obtained by CONTIN analysis of the correlation function embedded in Malvern software. The iron oxide content was determined using a PerkinElmer TGA 7 thermogravimetric analyzer (Norwalk, CT, USA). The PEGylated nanoparticles were heated from RT to 800 °C at 10 °C/min in air. ATR FTIR spectra were obtained on a PerkinElmer Paragon 1000PC spectrometer equipped with a Specac MKII Golden Gate single attenuated total reflection (ATR) system with a diamond crystal; the angle of incidence was 45°. Magnetization curves were measured at 300 K by an ADE Magnetics model EV9 VSM magnetometer (MicroSense; Lowell, MA, USA).

The grafting density (σ) of PEG on the particle surface was calculated according to Eq. (1) as follows:

1 $$\sigma =\frac{({m}_{PEG}/{m}_{Fe3O4}){\rho }_{core}\,V\,{{\rm{N}}}_{A}}{{M}_{{\rm{n}}}S}$$

where m_PEG_ and m_Fe3O4_ are the weight percentages of PEG and iron oxide in the particles according to TGA, respectively, ρ is the density of magnetite (5.18 kg/m^3^), N_A_ is Avogadro’s number, and *M*_n_ is the number-average molecular weight of PEG-Ner (2,165 g/mol).

The relaxivity (*r*_2_) of the magnetic nanoparticles was measured at 5–65 °C using a MiniSpec mq20 relaxometer (Bruker; Rheinstetten, Germany). The particle colloids were diluted to concentrations of 8–11 µg of Fe_3_O_4_/ml, and the *T*_2_ relaxation times were measured using a Carr Purcell Meiboom Gill (CPMG) multi-spin echo sequence (echo spacing *2τ* = 2 ms, repetition time *TR* = 5 s, and number of acquisitions *NA* = 8). The colloids were vortexed before each measurement. The relaxivity *r*_2_ was calculated as a reciprocal value of *T*_2_ divided by the nanoparticle concentration (after subtraction of the solvent contribution).

### *In vivo* biodistribution of Fe_3_O_4_@PEG-Ner particles in mice

#### Animals

C57BL/6NCrl mice were bred in a specific-pathogen free animal facility of the First Faculty of Medicine, Charles University (Prague, Czech Republic), and maintained in individually ventilated cages (12:12 h light-dark cycle, 22 ± 1 °C, 60 ± 5% humidity). The studies used adult male mice (6–8 weeks old) with free access to water and a standard rodent diet. The experiments were performed in accordance with national and international guidelines for laboratory animal care and approved by the Laboratory Animal Care and Use Committee of the First Faculty of Medicine, Charles University, and the Ministry of Education, Youth and Sports of the Czech Republic (MSMT-6316/2014-46).

#### MRI

The 10, 20, and 31 nm Fe_3_O_4_@PEG-Ner colloids (50 μl per animal) were intravenously injected into mice through the tail vein (each particle type was administered separately into different mice, and the control mouse was scanned without nanoparticle application). The animals were then subjected to MRI scanning using a 1 T ICON scanner (Bruker BioSpin; Ettlingen, Germany). The scanning protocol consisted of a localizer sequence (a fast gradient echo sequence providing images in three orthogonal slices with low resolution), a *T*_1_/*T*_2_*-weighted gradient echo sequence (echo time *TE* = 4 ms, *TR* = 160 ms, flip angle *FA* = 80°, *NA* = 16), and a strongly *T*_2_*-weighted gradient echo sequence (*TE* = 8 ms, *TR* = 400 ms, *FA* = 60°, *NA* = 8). The same geometry (1 mm coronal slices, 30 × 60 mm field of view, 128 × 256 matrix) was used for both sequences. The mice were anesthetized by spontaneous inhalation of isoflurane (5% for induction and 1.5% for maintenance) for both procedures (particle administration and MR scanning).

#### Assessment of nanoparticle circulation in blood

The 10, 20, and 31 nm Fe_3_O_4_@PEG-Ner colloids (50 µl per animal) were intravenously injected into the mice through the tail vein (each particle type was administered separately to three different mice, and two mice without particle administration were used as controls). Blood (100 µl) was obtained from the retro-orbital plexus at 5 and 30 min and 2, 4, 24, and 48 h after Fe_3_O_4_@PEG-Ner injection. Blood was collected in test tubes with the addition of sodium heparin to prevent coagulation. The *T*_2_ relaxation times were measured for each blood sample at 37 °C using a CPMG multi-spin echo sequence (*TE* = 2 ms, *TR* = 5 s, and *NA* = 8). The nanoparticle concentration (*c*) in blood was calculated from the nanoparticle relaxivities (*r*_2_) after the deduction of a contribution of the pure blood to the relaxation rate according to equation (2) as follows:

2 $$c=(1/{T}_{{\mathtt{2}}{\mathtt{b}}{\mathtt{N}}{\mathtt{P}}}\,{\textstyle {\mathtt{\text{-}}}}\,{\mathtt{1}}{\mathtt{/}}{{T}}_{{\mathtt{2}}{\mathtt{b}}}{\mathtt{)}}{\mathtt{/}}{{r}}_{{\mathtt{2}}}$$

where *T*_2bNP_ and *T*_2b_ are the relaxation times of the blood sample with nanoparticles and pure blood, respectively.

### MRI of Fe_3_O_4_@PEG-Ner nanoparticles in tumor-bearing mice

EL-4 (ATCC^®^ TIB-39™) mouse lymphoma cells were subcutaneously inoculated into C57Bl/6 mice (n = 4). Fe_3_O_4_@PEG-Ner-20 nanoparticles were intravenously administered in three doses (50 μl each) as follows: the first dose was administered one week after tumor implantation and the second and third doses were administered in 24 h intervals to prolong the blood circulation time. MRI was performed before nanoparticle administration (0 h time point), then 4 h after each particle administration, i.e., at 4, 28, and 52 h time points, and then at 120 and 192 h. The same MR scanning protocol was used as mentioned above.

## Results and Discussion

### Control of magnetic nanoparticle size

**Figure 1:**
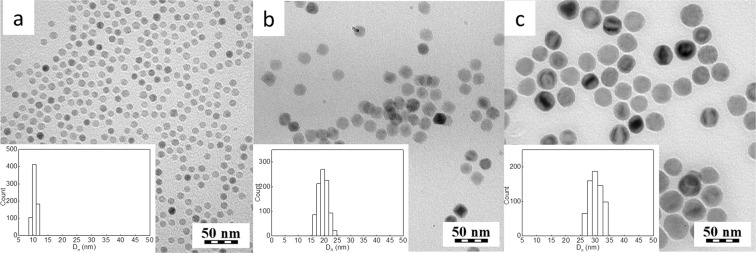
TEM micrographs of (**a**) 10, (**b**) 20, (**c**) 31 nm Fe_3_O_4_ particles prepared in (**a**,**b**) OD (320 °C) and (**c**) IS (343 °C). Insets show the particle size distribution histograms.

Uniformly-sized magnetic nanoparticles were prepared by the thermal decomposition of iron(III) carboxylates in high-boiling point solvents in the presence of OA as a stabilizer, which resulted in magnetite spinel phase, as confirmed by X-ray powder analysis^23^. The particle size was controlled by changing various reaction parameters, such as the type of iron precursor, amount of OA, reaction time and temperature. Decomposition of iron(III) oleate in OD (b.p. 320 °C) for 30 min in the presence of OA (4.55 g) produced 10 nm nanoparticles with *Ð* = 1.05 (Fig. 1a). The 20 nm (*Ð* = 1.03) Fe_3_O_4_ particles were obtained by decomposition of iron(III) mandelate under similar reaction conditions, but with a slightly lower amount of OA (4.4 g; Fig. 1b). In terms of morphology, the Fe_3_O_4_ particles were quite comparable with those reported earlier^16,35^. The variation in the particle size can be explained by different decomposition profiles of selected iron precursors. As shown previously, iron(III) mandelate decomposes faster; therefore, the nanoparticles nucleate and grow at lower temperatures than those from iron(III) oleate; as a result, larger particles were obtained^23^. Increasing the reaction temperature to 343 °C and prolonging the reaction time from 30 to 60 min during the decomposition of iron(III) mandelate resulted in the formation of large, 31 nm Fe_3_O_4_ particles with a narrow size distribution (*Ð* = 1.05). This formation could be attributed to an increased growth rate and a prolonged reaction time at an elevated temperature. According to TEM, the nanoparticles were almost spherical in shape (Fig. 1a–c).

### Engineering of the Fe_3_O_4_ surface with PEG-Ner

OA-coated magnetite nanoparticles are hydrophobic, which are not suitable for future biological use. Because the particles were injected in mice in physiological buffers, water-dispersible particles were required; therefore, the hydrophobic coating of the original particles needed to be exchanged with a hydrophilic coating. Iron oxide nanoparticles contain a large number of hydroxyl groups on the surface, which can be used for the covalent attachment of hydrophilic molecules. In this study, iron oxide nanoparticles with different sizes were modified with bis(phosphonic acid)-terminated PEG prepared from PEG-NHS and neridronate^26^, which is in contrast to commonly used alendronate^28^. PEG, which is a biocompatible and highly hydrophilic polyether that provides strong steric repulsions, is frequently used in medicine for the preparation of various pharmaceutical formulations^12,18,36^. The advantage of bisphosphonate groups is their strong affinity for metal ions, such as iron. Bisphosphonic acid-terminated PEG can thus be strongly bound to the Fe_3_O_4_ nanoparticle surface. Moreover, neridronate is biocompatible and has been approved for clinical use in the treatment of various bone illnesses^37^.

**Figure 2:**
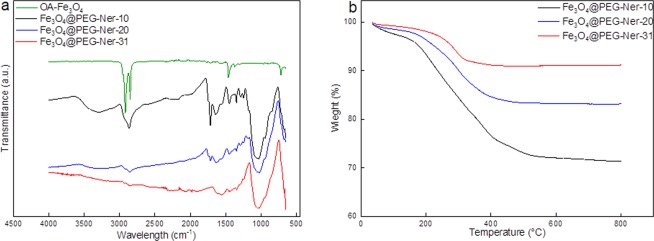
(**a**) ATR FTIR spectra of OA-coated Fe_3_O_4_ and Fe_3_O_4_@PEG-Ner-10-31 particles and (**b**) TGA of Fe_3_O_4_@PEG-Ner-10-31 nanoparticles.

Fe_3_O_4_@PEG-Ner particles were thoroughly characterized by a range of physicochemical methods, including DLS, FTIR spectroscopy, thermogravimetric analysis (TGA), and magnetization measurements. The ATR FTIR spectra of all Fe_3_O_4_@PEG-Ner particles were compared with that of OA-coated Fe_3_O_4_ (Fig. 2a). While the 10-nm OA-coated particles exhibited peaks at 2,920, 2,851, and 1,466 cm^−1^ assigned to the ν_as_(CH_3_), ν_as_(CH_2_) asymmetric, and ν(C=O) stretching vibrations, respectively, originating from OA, the strong band at 1,050 cm^−1^ attributed to the vibration of the C-O-C group confirmed the presence of PEG on the Fe_3_O_4_@PEG-Ner surface. Let us to note that the ATR FTIR spectra of 20- and 31-nm OA-coated particles resembled that to the 10-nm one (data not shown). According to TGA, the amount of PEG on the nanoparticle surface decreased with increasing particle size, reaching 29, 17, and 9 wt.% for 10, 20, and 31 nm particles, respectively (Table 1, Fig. 2b).

**Table 1:** Characterization of the Fe_3_O_4_@PEG-Ner nanoparticles.

Particles	*D*_n_ (nm)	*Ð*	*D*_h_ (nm)		*PI*		ζ-potential (mV)		Coating^a^ (wt.%)	σ^b^ (PEG/nm^2^)	*M*_s_^c^ (Am^2^/kg)	*M*_r_^d^ (Am^2^/kg)
			Water	PBS	Water	PBS	Water	PBS				
Fe_3_O_4_@PEG-Ner-10	10	1.05	35	34	0.2	0.26	−9	−6	28.5	0.99	34	0
Fe_3_O_4_@PEG-Ner-20	20	1.03	40	39	0.1	0.16	−14	−3	17	1.02	40	0.2
Fe_3_O_4_@PEG-Ner-31	31	1.05	65	62	0.14	0.11	−15	−3	9	0.76	42	7

*D*_n_ - number-average particle diameter (TEM); *Ð* - dispersity (TEM); *D*_h_ - hydrodynamic diameter (DLS); *PI* - polydispersity (DLS); ^a^amount of PEG-Ner (TGA); ^b^grafting density; ^c^saturation magnetization; ^d^remanent magnetization.

Different amounts of PEG on the particles can be explained by the larger surface-to-volume ratio of the 10 nm particle than those of the 20 and 31 nm particles, resulting in an increased amount of bound PEG on the smallest particles. The highest PEG grafting density was found on the 20 nm particles (1.02 PEG/nm^2^), while the lowest density was found on the 31 nm particles (0.76 PEG/nm^2^; Table 1). In theory, if the molecular weight and surface density of PEG are known, it is possible to predict its conformation^38^. The grafting density controls the conformation of PEG chains on the surface, which can be brush-like or mushroom-like. Generally, the mushroom-like conformation is observed at low surface density when *d* > *r*_f_, where *d* is the distance between the attachment points of the polymer on the surface and *r*_f_ is the Flory radius. Furthermore, *d* < *r*_f_ indicates a brush-like conformation^39^. The *r*_f_ was 3.44 nm for PEG and *d* was 1, 0.99, and 1.15 nm for Fe_3_O_4_@PEG-Ner-10, -20, and -31, respectively. The fact that *d* was smaller than *r*_f_ in all PEGylated nanoparticles confirmed dense packing of PEG on the surface in a brush-like conformation. This organization of the shell is beneficial for the application of magnetic nanoparticles in medicine because it could significantly decrease protein adsorption and recognition by macrophages and prolong particle circulation in the blood stream^40^.

**Figure 3:**
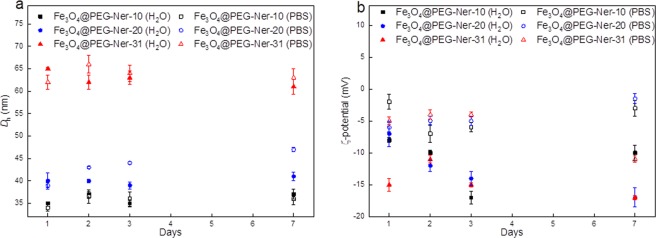
Time dependence of (**a**) hydrodynamic diameter *D*_h_ and (**b**) ζ-potential of nanoparticles in water and PBS (pH = 7.4).

**Figure 4:**
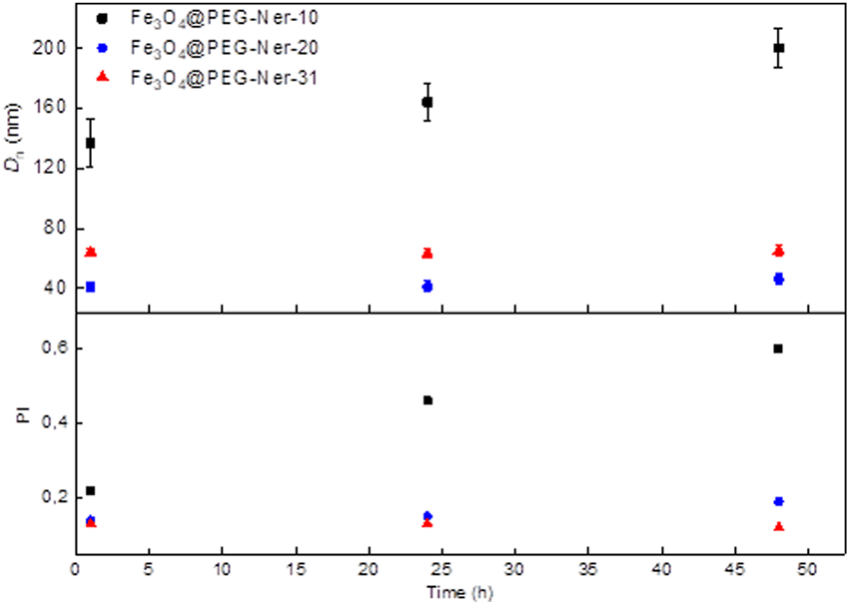
Time dependence of hydrodynamic diameter *D*_h_ and polydispersity *PI* of nanoparticles in PBS (pH = 7.4) in the presence of BSA (0.5 mg/ml).

The hydrodynamic diameters (*D*_h_) of Fe_3_O_4_@PEG-Ner-10, -20, and -31 were 35, 40, and 65 nm in water and 34, 39, and 62 nm in PBS (pH 7.4), respectively (Table 1). The polydispersity (*PI*) was 0.2, 0.1, and 0.14 in water and 0.26, 0.16, and 0.11 in PBS for Fe_3_O_4_@PEG-Ner-10, -20, and -31 nanoparticles, respectively. The difference in *D*_h_ and *D*_n_ (the size of the dried particles obtained by TEM) was attributed to the binding of PEG to the particle surface. Relatively similar *D*_h_ values of the particles in both media confirmed that the particles possessed good colloidal stability. The particle ζ-potential ranged from −9 to −15 mV and from −3 to −6 mV in water and PBS, respectively. A relatively low negative surface charge was likely caused by the presence of phosphonate groups. Monitoring of the magnetic nanoparticles in both water and PBS for one week showed no significant changes in the *D*_h_ or ζ-potential, indicating excellent longtime colloidal stability that is crucial for biomedical applications (Fig. 3a,b). This observation also suggests that the particles were mainly stabilized by strong steric repulsions of PEG chains on the particle surface, which, in contrast to electrostatic repulsions, is insensitive to the presence of different ions. In PBS containing BSA (0.5 mg/ml), the *D*_h_ and polydispersity of Fe_3_O_4_@PEG-Ner-10 particles increased after 1 and 48 h to 137 and 200 nm and *PI* to 0.22 and 0.6, respectively (Fig. 4). Interestingly, the *D*_h_ and *PI* of both Fe_3_O_4_@PEG-Ner-20, and -31 particles did not change with time and the sizes were similar to those in neat PBS, confirming that the surface was well protected by PEG. Rapid increase of hydrodynamic Fe_3_O_4_@PEG-Ner-10 nanoparticle size with time was attributed to protein corona formation due to higher ζ-potential. The saturation magnetization (*M*_s_) of Fe_3_O_4_@PEG-Ner-10, -20, and -31 particles was 34, 40, and 42 Am^2^/kg, respectively (Table 1). The *M*_s_ values were lower than that of the bulk magnetite (*M*_s_ = 92–100 Am^2^/kg)^41^, which can be attributed to different surface-to-volume ratios, spin-canting effects, and surface anisotropy disorder^42–45^. Nevertheless, the magnetic properties of studied particles were comparable with those published elsewhere, where *M*_s_ = 51 and 39.7 Am^2^/kg^12,27^, and enabled their efficient and quick manipulation by an external magnetic field; moreover, they were satisfactory for application of the nanoparticles as a MRI contrast agent *in vivo*. The absence of remanent magnetization (*M*_r_) in the 10 nm particles or the negligibly small *M*_r_ of the 20 nm particles confirmed their superparamagnetic character. In contrast, the 31 nm nanoparticles were ferrimagnetic with *M*_r_ = 7 Am^2^/kg (Table 1).

### MR relaxivity of Fe_3_O_4_@PEG-Ner particles

**Figure 5:**
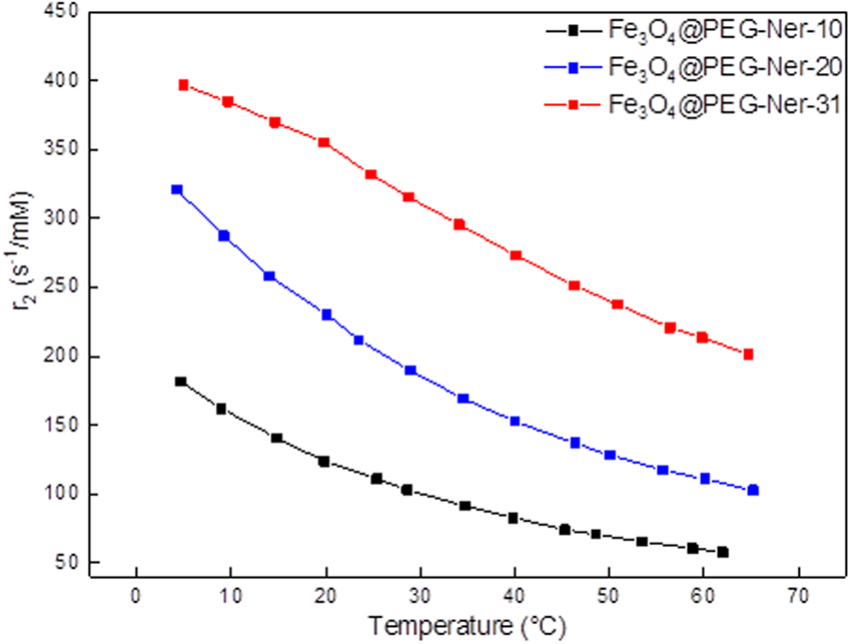
Dependence of relaxivity *r*_2_ of Fe_3_O_4_@PEG-Ner nanoparticles on temperature at 0.5 T.

As expected, the relaxivity (*r*_2_) of diluted Fe_3_O_4_@PEG-Ner-10-31 colloids measured *in vitro* at 0.5 T magnetic field decreased with increasing temperature ranging from 5–65 °C (Fig. 5). This behavior was not surprising in the so-called motional averaging regime of the small particles or the static dephasing regime in the large particles^46^ when the temperature dependence of water diffusion and water exchange on the particle surface was considered^47^. The Fe_3_O_4_@PEG-Ner-10 nanoparticles exhibited the lowest relaxivity due to their smaller saturation magnetization. Relaxivities (reciprocal values of relaxation times related to Fe_3_O_4_ crystal unit) of the particles corresponded to already published results with respect to their size and used magnetic field. Some differences can be caused by different coatings^48–50^.

### Fe_3_O_4_@PEG-Ner nanoparticle biodistribution in mice assessed by MRI

**Figure 6:**
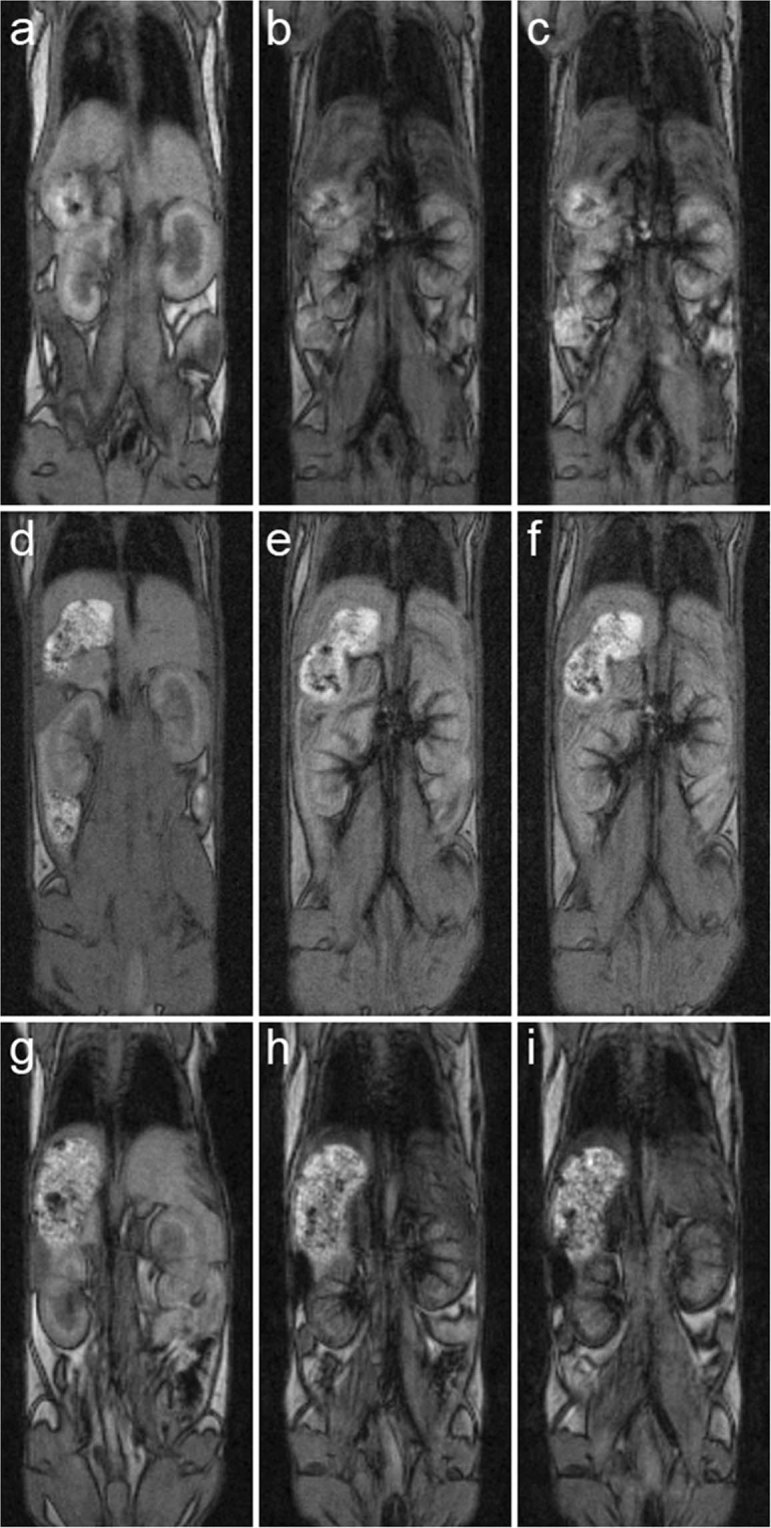
MRI coronal images of mice (**a**,**d**,**g**) before application, (**b**,**e**,**h**) 10 min and (**c**,**f**,**i**) 60 min after application of (**a**–**c**) Fe_3_O_4_@PEG-Ner-10, (**d**–**f**) Fe_3_O_4_@PEG-Ner-20, and (**g**–**i**) Fe_3_O_4_@PEG-Ner-31 particles. High nanoparticle concentration in the blood after administration was manifested by a strong hypointense signal in the veins, clearly visible in the kidneys. Note also strong hypointense signal of (**b**,**c**) Fe_3_O_4_@PEG-Ner-10 and (**g**,**h**) Fe_3_O_4_@PEG-Ner-31 in the liver caused by faster clearance via the liver and weak hypointense signal of (**e**,**f**) Fe_3_O_4_@PEG-Ner-20 in the liver caused probably by slower clearance and thus prolonged blood circulation time. Images were obtained by a gradient echo sequence with weak *T*_2_* weighting.

With the aim to find an optimal size for a prolonged blood stream circulation, newly synthesized 10-, 20-, and 31-nm particles were tested in experimental animals. MRI confirmed the presence of the Fe_3_O_4_@PEG-Ner nanoparticles in the blood stream of the mice after intravenous injection, which was indicated by a hypointense signal in the vessels and tissues that are well-perfused by blood. The *in vivo* distribution of the nanoparticles depended on the particle size. As the nanoparticles were cleared from the blood stream through excretion by the hepatobiliary route, the hypointense signal was observed mainly in the liver tissue (Fig. 6). Interestingly, a strong hypointense signal in the liver shortly after administration (10 and 60 min) was observed for the Fe_3_O_4_@PEG-Ner-10 and -31 particles, whereas the Fe_3_O_4_@PEG-Ner-20 nanoparticles provided a weak signal, likely because their clearance was slower and the blood circulation time was longer. Four hours after administration, a strong hypointense signal in the liver was observed, even for Fe_3_O_4_@PEG-Ner-20 nanoparticles. It corresponded to typical final biodistribution of iron oxide nanoparticles (80–90% in liver, 5–8% in spleen, and 1–2% in bone marrow)^7^. It was reported, that clearance of magnetic nanoparticles from liver can be longer than 14 days^51,52^. A noticeable hypointense signal was also observed in the kidneys due to high blood perfusion of this organ. However, certain entrapment of the nanoparticles in the kidney tissue could not be excluded.

### Circulation of Fe_3_O_4_@PEG-Ner nanoparticles in blood

**Figure 7:**
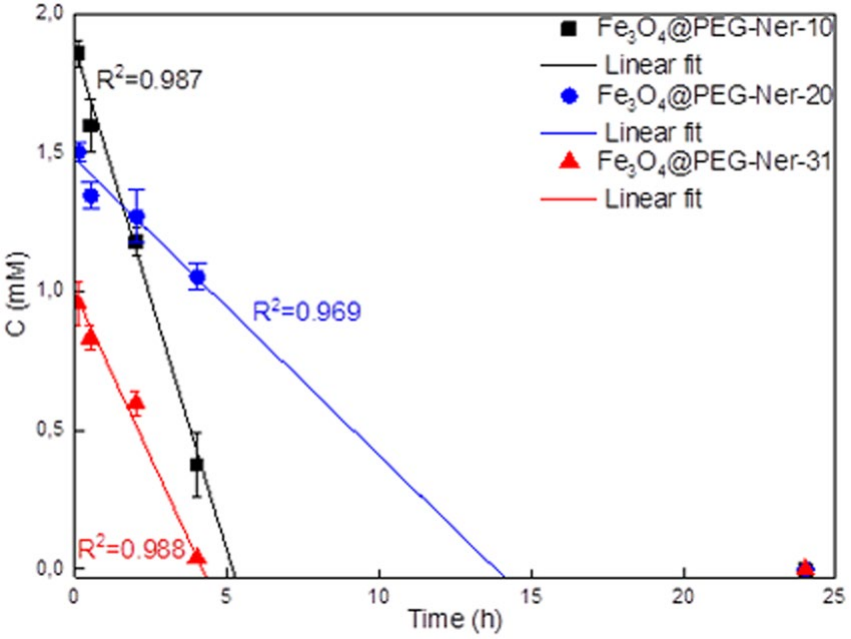
Time dependence of Fe_3_O_4_@PEG-Ner particle concentration in mice blood after intravenous injection.

The concentration of the Fe_3_O_4_@PEG-Ner nanoparticles in the blood of experimental animals was determined by *T*_2_ relaxometry measurements (Fig. 7). Notably, the reduction in Fe_3_O_4_ concentration (for all three particle sizes) in the blood did not follow an exponential course but was rather linear, which suggests that the elimination of the particles did not solely depend on their blood concentrations. Non-exponential elimination contradicts some already published data^49^, which showed monoexponential decay of iron oxide concentration. However, these particles were coated with carboxydextran or poly(acrylic acid). Carboxydextran-coated particles have circulation half-time in the order of minutes^50^. PEG coating used in our study prolonged the circulation of particles to hours by decreasing macrophage uptake, which is in agreement with previous reports^53^. This effect might influence the kinetics of particle elimination. In fact, the speed of elimination might have also been affected by the liver capacity on the cellular level and the linear dependence indicates that liver capacity was saturated. The Fe_3_O_4_@PEG-Ner-10, -20, and -31 particles were removed from the blood stream within 5, 14, and 4 h, respectively, and no nanoparticles were detected in the blood at 25 h postinjection. The results corresponded to the *in vivo* MRI results, which indicated slower excretion of the 20 nm particles by the liver. This trend was in contrast with the excretion of the commercial MRI contrast agent Resovist^®^ (carboxydextran-coated iron oxide) that is immediately engulfed by the liver. Similarly, blood half-life of dimercaptosuccinic acid-coated iron oxide particles was only 3 min for dispersions with *D*_h_ = 65–70 nm and 15 min for those with *D*_h_ = 30 nm^34^. Fe_3_O_4_@PEG-Ner-20 nanoparticles had also longer blood circulation time than poly(maleic anhydride-*alt*-1-octadecene)-PEG- and bisphosphonate PEG-coated iron oxides with 25 and 6 nm core size, respectively^27,30^.

Generally, the circulation of nanoparticles in the blood is influenced by many factors. For example, it depends on retention in different organs, primarily the liver, which typically represents the main route of iron oxide elimination. The circulation time may also be related to the hydrodynamic diameter. Relatively long circulation times were found for Fe_3_O_4_@PEG-Ner-20 nanoparticles (14 h) compared to those of other particle sizes. Although the hydrodynamic size and density of the PEG chains grafted to the Fe_3_O_4_@PEG-Ner-10 and Fe_3_O_4_@PEG-Ner-20 particle surfaces were similar (Table 1), the former particles had a shorter circulation time, which could have been due to their tendency to aggregate and a lower negative ζ-potential in PBS, leading to increased opsonization and faster elimination from the blood stream. This observation is in agreement with an earlier report describing the interaction of β-2 glycoprotein and apolipoprotein B with negatively charged surfaces^54^. Both of these liver proteins are able to attach to foreign substances and transport them *in vivo* by the lymphatic system or blood^55^. They also adhere to negatively charged nanoparticles, transferring them into the liver. Regarding the Fe_3_O_4_@PEG-Ner-31 nanoparticles, their PEG density was the lowest of the investigated samples, and the blood circulation time was short. Therefore, it can be speculated that the relatively high PEG density of Fe_3_O_4_@PEG-Ner-20 nanoparticles formed a more stable coating than that in Fe_3_O_4_@PEG-Ner-31 particles, which improved the separation of the core from the outer environment and led to slower elimination of the nanoparticles by the liver. Long circulation of the Fe_3_O_4_@PEG-Ner-20 nanoparticles was thus probably given by superior combination of PEG-neridronate coating and proper size of the particles. Moreover, protein corona (both soft and hard) formed on nanoparticles in the blood stream can strongly affect their behavior in biological systems, in particular, interaction with platelets and blood cells, which in turn influences removal of the particles from blood^56^. Although the fast elimination of the contrast agent is typically desirable in clinical practice to minimize possible adverse effects, prolonged particle circulation might be advantageous for imaging organs or structures requiring longer exposure for the entrapment of the contrast agent.

### MRI of Fe_3_O_4_@PEG-Ner nanoparticles in tumor bearing mice

**Figure 8:**
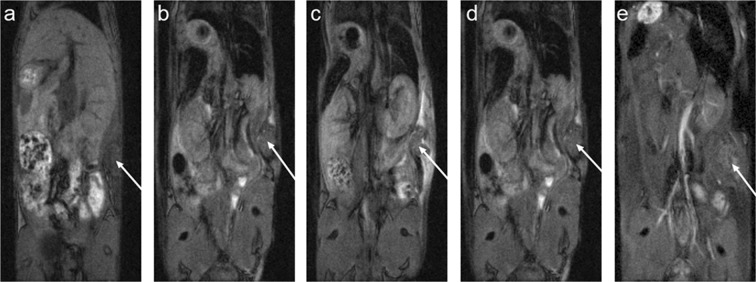
MRI of a mouse with EL-4 tumor (arrow shows its location). (**a**) MRI at time points 0 (before nanoparticle application), (**b**) 4 h (4 h after the first dose of 50 μl of Fe_3_O_4_@PEG-Ner-20 particles), (**c**) 28 h (4 h after the second dose), (**d**) 52 h (4 h after the third dose), and (**e**) 120 h (72 h after the third dose). Note the growth of the tumor during the experiment.

**Figure 9:**
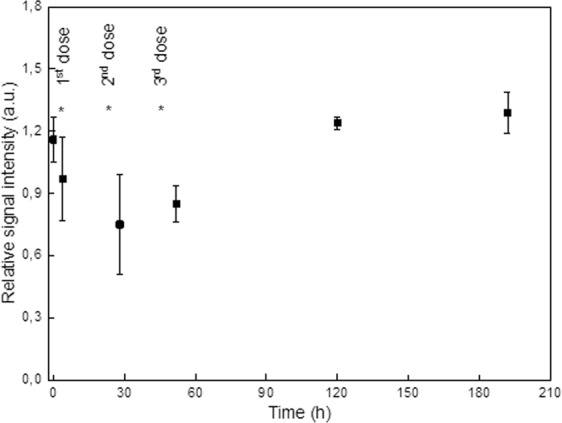
MRI of mice with EL-4 tumor. Dependence of relative signal intensity of the contrast in the tumor on time after the first Fe_3_O_4_@PEG-Ner-20 particle application. The asterisks show time points of nanoparticle administration. Signal intensity at time points 28 and 52 h (i.e., after the second and third nanoparticle administration) are significantly lower (t-test, p < 0.05) than that at time 0 (before nanoparticle administration) or at time points 120 and 192 h.

Finally, the Fe_3_O_4_@PEG-Ner-20 nanoparticles with the longest blood circulation time were intravenously injected into mice with an induced subcutaneous tumor, and MRI scans were obtained (Fig. 8). A hypointense signal was observed in the liver 4 h after particle administration, whereas the signal decrease in the tumor was insignificant. The particles were then administered repeatedly after 24 and 48 h to prolong the blood circulation time and enable retention of the particles by the enhanced permeability and retention (EPR) effect. Although a small but significant signal decrease was observed in the tumor shortly after the second and third administrations (Fig. 9), the nanoparticles were quickly eliminated. No signal change was detected compared to the precontrast examination three days after the injection of the final dose, which indicated low retention of the nanoparticles in the tumor.

Cleary, the EPR effect was not observed in our study, which is in agreement with clinical practice, where tumor targeting via EPR has not been proved in humans^57^. This is the reason why intratumoral injections are generally preferred to achieve an accumulation of the particles in a tumor. In addition, the internalization of PEG-coated particles in the cells may be hindered by the nonionogenic nature of PEG and its steric repulsions and antifouling properties^58^. Moreover, biological applications of PEG-modified particles were recently questioned due to enhanced serum protein binding and a significant reduction in drug delivery^59^. Other studies have also reported on the immunogenicity of PEG^60^, which requires the development of innovative coatings and/or targeting moieties for blood delivery systems in the near future.

## Conclusion

Magnetite nanoparticles with a controlled size and uniform size distribution were prepared by thermal decomposition of Fe(III) oleate and mandelate in nonpolar high-boiling point solvents. According to TEM, the Fe_3_O_4_ size was 10, 20, and 31 nm, with dispersity *Ð* = 1.05, 1.03, and 1.05, respectively, indicating a very narrow particle size distribution. In the literature, such particle sizes were not yet investigated in terms of their biodistribution in an animal. Particle size uniformity is important in biomedical applications, which require high quality particles with identical physical, chemical, and biological properties to achieve reproducibility of the results. The Fe_3_O_4_ surface was hydrophilized with PEG-containing bisphosphonate anchoring groups to prevent particle aggregation. In contrast to commonly used PEG-alendronate, the particles were modified by novel PEG-neridronate, which rendered them with superior colloidal stability in physiological media. According to the thermogravimetric analysis, the amount of the particle coating decreased with increasing particle size, which was attributed to the different surface-to-volume ratios of the particles. The calculations showed that the 10, 20, and 31 nm particles were coated with brush-like PEG shells with grafting densities of 0.99, 1.02, and 0.76 PEG/nm^2^, respectively. The *D*_h_ of the Fe_3_O_4_ particles ranged from 34 to 62 nm in PBS (pH 7.4), which is ideal for biomedical applications^61^. In addition, the *D*_h_ was nearly constant in water and PBS for one week, confirming the superior colloidal stability of the particles that was maintained by the steric repulsion of PEG. The particle relaxivity increased with increasing size. Fe_3_O_4_ clearance from the blood stream in the mouse model exhibited a linear time dependence, which was attributed to the uniform particle size that provided the same properties. The particle circulation time was attributed to the Fe_3_O_4_ size and the quality of the surface coating. The *in vivo* elimination of the Fe_3_O_4_ particles depended on the PEG density on the particle surface and the ζ-potential rather than the particle size. Because the PEG density was high on the Fe_3_O_4_@PEG-Ner-20 particles, they circulated in the blood for the longest period of time out of all the investigated samples.

In a mouse tumor model, the majority of the Fe_3_O_4_@PEG-Ner-20 nanoparticles were taken up by the liver after intravenous injection. Significant retention of the particles in the tumor was not achieved, even with repeated administration. Therefore, alternative strategies for PEG-modified particles must be investigated to prolong blood circulation times and/or to achieve specific targeting in a tumor.
